# After Myriad: Genetic Testing in the Wake of Recent Supreme Court Decisions about Gene Patents

**DOI:** 10.1007/s40142-014-0055-5

**Published:** 2014-09-11

**Authors:** Robert Cook-Deegan, Annie Niehaus

**Affiliations:** 1 Sanford School of Public Policy, Duke University, Durham, NC USA; 2 Pomona College, Claremont, CA USA

**Keywords:** Patents, Genetic testing, Supreme Court, Biotechnology, Law

## Abstract

Genetic testing is becoming more common and more powerful by the day. The costs of the underlying DNA sequencing technology are plummeting, making it likely that tests based on it will become even more pervasive. The use of tests to determine DNA sequence to help make clinical decisions is here to stay. DNA sequencing is also finding new uses in forensics, determination of ancestry, understanding the history and genetic lineages of human populations and many other applications.

## Background

Clinical “genetic tests” span a wide range of methods and uses. One common element is using DNA to infer information about the sequence of DNA or RNA in a blood or saliva sample, or from another biological sample taken from a person. Such testing can be either to determine that person’s genetic inheritance (genotype), for example to predict risk of developing a disease that can be inherited. Genetic testing can also be done on samples of a tissue or tumor to identify changes that have occurred in cells in that person’s body (somatic cell testing), for example to find DNA changes that have developed in cancer cells.

The general approach is to correlate structural change in molecules with clinical outcomes, in a field often described as “molecular diagnostics,” although some uses are not strictly speaking just for diagnosis, but also for prognosis, identification of perturbed molecular pathways, guiding selection of treatments, or determination of cellular origin. DNA-based testing is as subset of molecular diagnostics. Molecular diagnostics is broader, encompassing proteins, lipids, carbohydrates, drug or nutritional metabolites, and other constituents of cells. This paper concerns only DNA-based tests, and concerns only patents on individual genes.

Diagnosis and prognosis in clinical medicine are the foremost and, to date, most lucrative early applications of genetic and genomic technologies. Clinical genetic testing is often the first practical use of gene-based discoveries, once a gene is cloned and characterized. The prospect of commercial genetic testing is a major source of both public and private investment. Clinical genetic testing constitutes a common pathway to widespread use of genomics, often including commercialization of DNA-based tests. These medical uses of genetic testing are affected by many policies, including patent policy, regulation, and the coding, coverage, and reimbursement of genetic tests.

### The Supreme Court Reins in What Can be Patented

This article addresses just one policy domain where changes have attracted considerable attention: patents on individual human genes. Several prominent recent cases have demonstrated clearly how decades of conventional wisdom about what is and is not patent-eligible was wrong at the margin, at least in the United States. It turns out that lawyers confident about the boundaries of what can be patented in biotechnology have been giving errant advice to their clients, based on understandings about patent rules that the US Supreme Court has decisively repudiated.

While the Court has been clear that DNA molecules and methods hitherto assumed to be patent-eligible are not, it has been far from clear in explaining how to draw the line between what can be patented and what cannot. The Court unanimously invalidated patent claims that many in the patent bar assumed were valid claims. That is, the Court has made clear that some discoveries that many assumed were patentable are not, including DNA molecules whose sequences are found in nature. It has not, however, made clear how much human intervention is needed to convert unpatentable discoveries into patentable inventions.

Changing jurisprudence is now driving changes in how researchers, research institutions, and companies engaged in genetic testing make decisions about whether, when, and how to patent discoveries and inventions. The business of patenting genes now faces considerable uncertainty, not because the Court has changed the rules, but because those in the patent game had gotten used to a looser set of rules and now have to adjust to the rules the Supreme Court asserts should have prevailed all along, but without much guidance about how to do so.

To understand the nature and cause of that uncertainty, the history of two recent cases that reached the Supreme Court is illuminating. The account follows the cases that were unanimously decided by the Supreme Court in 2012 and 2013. Following those decisions through the US federal court system conveys the complexity of gene patent policy, and how hard judges have to work to understand the legal, technical, medical, and economic stakes affected by the decisions they are making. The patent cases read like roller-coasters, with ups and downs and many turns. The purpose of laying out the details is not just interesting history, but to show how much uncertainty still pervades patent policy relevant to genetic diagnostics.

The main upshot of the analysis, however, is clear: the Supreme Court has repeatedly and unanimously signaled that patent rights were being granted for unpatentable subject matter—allowing claims on methods that were too broad and on DNA molecules that corresponded to sequences found in nature. In that respect, the law is now clear: such claims will be ruled invalid. The recent decisions should thus reduce the shadow of uncertainty cast over exome sequencing and whole-genome sequencing. That shadow was caused by patent claims that were granted but appeared to be infringed by *any* means of making DNA molecules or determining DNA sequence of the genes being claimed [see box A]. Infringement would pertain to any form of whole-genome analysis that included the patented gene, because in doing such analysis, one would make DNA molecules from fragments of the patented individual gene as described in the claims, use methods claimed in individual gene patents, or do both. The recent court rulings thus clear the path for unfettered pursuit of whole-genome analysis and multi-gene methods, although the exact extent of patent protection conferred by individual gene patents is still being defined by ongoing litigation that began in July 2013, a month after the Supreme Court decision in *Myriad.*

### Interaction between Patents, FDA Regulation, and Coverage and Reimbursement

The main role of patents in biotechnology and medical applications is to induce private investment in research and development (R&D). Most genetic discoveries themselves—efforts to clone and characterize disease-associated genes or to unravel metabolic pathways, for example—are based on publicly funded research conducted at hospitals, non-profit research institutes, or academic health centers. Such discoveries are frequently patented, but the patents do not generally add strong incentives for the discovery of genes; rather the patents are bundles of rights that can be licensed to firms to develop into commercial products and services post-discovery.

Only occasionally does private R&D lead to initial gene discoveries; the patent incentive is relatively weak as a “pull” for initial discovery. The *BRCA1* gene associated with breast cancer and the *HFE* gene associated with hemochromatosis were the only genes discovered by private firms among case studies of dozens of genes underlying ten clinical conditions prepared for the Secretary’s Advisory Committee for Genetics Health and Society [1]. This led the Committee to conclude that “patent-derived exclusive rights are neither necessary nor sufficient conditions for the development of genetic test kits and laboratory-developed tests” [2•, p. 35].

The Committee also noted, however, that patents might become more important if the Food and Drug Administration (FDA) begins to more directly regulate laboratory-developed tests, or if payers require clinical studies to demonstrate clinical utility before covering and reimbursing genetic tests. FDA premarket approval would make test development far more expensive than just setting up an assay, and could warrant patent protection. The relevant patents, however, are unlikely to be on individual genes. Patents on sets of genes or specific methods for measuring DNA changes could interact with FDA regulation as well as coverage and reimbursement policy. A patent on a particular FDA-approved test for multiple alleles, for example, might provide exclusivity on that particular set of measurements, and a competitor would not have FDA approval to market the test if it made any significant modifications (e.g., changing the genes assayed or using a different interpretive algorithm); in this way, the patent on a method or set of molecules (but not individual genes) could provide meaningful exclusivity even as individual genes are not patent-eligible. This strategy is still open despite the Supreme Courts rulings described below. The interaction between patents, regulation, coding, and insurance coverage and reimbursement are intricate and beyond the scope of this review, except to note they merit a separate analysis in greater depth.

## Shifting Sands of Patent Jurisprudence

Patents on genes associated with human disease have been controversial since they began to be granted. Patents are government-conferred rights to exclude others from making, using, selling, or importing the invention claimed. They have two parts, a description (or specification) of the invention and a set of claims that define the boundaries of the intellectual property. Patent litigation generally centers on the exact language of patent claims.

The role of patents in diagnostics generated much more public comment and conflict than patents on genes encoding known proteins with therapeutic benefit. The early use of recombinant DNA methods to produce protein therapeutics such as insulin, growth hormone, tissue plasminogen activator, interferon, and erythropoietin included patents on the genes encoding them [3]. Early gene patents were granted to “gene jockeys” who cloned and expressed therapeutic proteins of clinical value. Patents were granted in all major jurisdictions, although how they were interpreted and used differed among the United States, Europe, Japan, and other jurisdictions. Patents on genes that encoded therapeutic proteins led to patent litigation and legal conflict, but that was largely confined to battles among competing companies, rather than public controversy.

Public controversy arose far more in the context of diagnostic use of patents granted to those who discovered and characterized disease-associated genes. Policy reports on “gene patents” began to appear in the early to mid-1990s, an indicator of emerging policy conflict [4]. The discovery of genetic changes associated with Huntington’s disease, Duchenne and Becker muscular dystrophy, cystic fibrosis, neurofibromatosis, Alzheimer’s disease and other conditions led to DNA-based diagnostic methods to identify those at high-inherited risk in families with apparent Mendelian inheritance patterns. A patent on the *ASPA* (aspartoacylase A) gene was granted to Miami Children’s Hospital, and caused a rift and a lawsuit between the Hospital and the families and organizations that had enabled the discovery but disagreed with the Hospital’s commercialization strategy [5, 6]. That lawsuit did not challenge the patent directly, however, but alleged breach of informed consent and unjust enrichment.

### Early BRCA Patent Litigation

The patenting of the *BRCA1* and *BRCA2* genes, on chromosomes 17 and 13, respectively, bred by far the most conspicuous and intense controversy in gene patent policy. *BRCA* testing was mentioned far more often than any other case in policy reports on gene patent policy [4], and coverage in general newspapers in English-speaking countries was overwhelmingly negative [7], even before it became the subject of high-profile litigation in 2009.

Litigation over *BRCA* patents began in 1997 [8]. OncorMed sued Myriad Genetics alleging infringement of a patent on the consensus sequence of *BRCA1* granted to OncorMed in August. Myriad countersued the day after it got its first *BRCA1* patent in December. Myriad also filed (but never fully served) a lawsuit against the University of Pennsylvania. The University quickly agreed not to do testing except for its own patients; OncorMed and Myriad settled out of court on terms that were not made public [9]. These legal skirmishes attracted some media attention, but the real controversy swirling around Myriad Genetics and its *BRCA* gene patents centered on its business model and the way it used its patent rights [8, 9].

Nine laboratories that had been offering BRCA testing withdrew from the US market [10], and no one challenged Myriad’s patents for a decade. Myriad Genetics became the only commercial *BRCA* testing laboratory in the United States, although its service monopoly did not become established in any other country [9, 11, 12]. Through 2013, Myriad performed over one million *BRCA* tests and generated over $2.8 billion in revenues.^1^

### *Mayo v Prometheus*: a Supreme Court Decision about Diagnostic Method Claims

The stage for the famous *Myriad* case that went to the Supreme Court was set by several other cases bearing on diagnostics, although not specifically DNA diagnostics. In 2006, the Supreme Court agreed to hear, but then ultimately decided not to rule on *LabCorp v Metabolite.* The patent in question was on a test measuring levels of homocysteine to evaluate the likelihood of deficiencies in folate and cobalamin (vitamins B6 and B12). The Supreme Court’s finding was one terse sentence saying its grant of appeal had been “improvidently granted,” and declining to rule on the case. The only explanation and extended prose was a spirited dissent written by Justice Breyer, joined by Justices Souter and Stevens. The dissenters invited a future case that would center on diagnostic methods that depended on correlations and raised questions of patentable subject matter [13].

Another case seeming to partly answer Justice Breyer’s invitation came several years later in the form of *Mayo v Prometheus.* The patent that Mayo challenged in that case involved a method for measuring metabolites to adjust the dose of thiopurine drugs (used as anti-inflammatory treatments). The Court of Appeals for the Federal Circuit had twice upheld claims on the patented method. The Supreme Court ruled unanimously that the claims were invalid because the method reached to a law of nature. This ruling had implications for genetic diagnostics, because method claims were often the broadest and hardest to work around in gene patents [14–16]. The *Mayo* ruling meant that many of the claims granted on individual genes, where they covered any way of measuring a sequence variant, would be ruled invalid if challenged.

### The BRCA Case that Went to the Supreme Court

Even as *Mayo* was making its way through the courts, the legal landscape was changing on another front, this time centered specifically on DNA molecules (as opposed to methods). In May 2009, the American Civil Liberties Union (ACLU) and Public Patent Foundation filed suit against Myriad Genetics, the US Patent and Trademark Office and the University of Utah [17]. That suit, filed in New York’s Federal District Court in Manhattan, followed a two-year process of deliberation within ACLU to decide whether and how to directly challenge human gene patenting as a matter of public interest law.^2^ ACLU gathered 20 plaintiffs: women wanting to get tested, physicians wanting to order tests, laboratory directors wanting to offer BRCA testing, and organizations [18]. The Association for Molecular Pathology was first to agree to sign onto the suit, and became the lead plaintiff.

### Federal District Court Decision

In a remarkable happenstance, the case was assigned to Judge Robert Sweet, who at the time had a clerk, Herman Yeu, with a background in molecular biology and a PhD from the University of California, Berkeley [19]. In March 2010, Judge Sweet rocked the patent world by ruling that all fifteen claims in the seven Myriad patents challenged in the lawsuit were invalid. He did this via summary judgment (without a trial) as a matter of law, basing his decision on Section 101 of the US patent statute that defines what can be patented. That is, he ruled that the claims did not clear the threshold of patentable subject matter, without even getting to the factual questions about other patent criteria: novelty, utility, nonobviousness, enablement and written description [20•].

There were three basic classes of claims. Some claims were on DNA molecules constituting the gene encoding BRCA1 and BRCA2 proteins; others were methods of detecting alterations in the sequence; and one challenged claim was for a method of using *BRCA1* as part of a cancer drug assay. Judge Sweet found that the claims on DNA molecules were invalid because “DNA represents the physical embodiment of biological information, distinct in its essential characteristics from any other chemical found in nature” and therefore unpatentable [20•] (at pp. 2–3). He rejected the argument that the DNA molecules claimed were patentable because they were “isolated,” noting that some called that a “lawyer’s trick.” As to the method claims, he judged “the claimed comparisons of DNA sequences are abstract mental processes [that] constitute unpatentable subject matter.”

### Court of Appeals for the Federal Circuit Decisions

Myriad appealed Judge Sweet’s district court decision to the Court of Appeals for the Federal Circuit (CAFC), a special court that Congress established in 1982. CAFC hears patent appeals from all district courts. The case went to a three-judge panel of Alan Lourie, Kimberly Moore, and William Bryson. Judge Lourie wrote the majority opinion that had four major components: (1) it concurred with judge Sweet’s invalidation of five of the six method claims; (2) it held that only one plaintiff, Harry Ostrer, had standing to sue; (3) it upheld the one method claim on use of *BRCA1* in a cancer drug assay; and (4) it reversed Judge Sweet to uphold nine claims on DNA molecules. The first three components were unanimous.

On the fourth point—whether the DNA molecule claims were patentable subject matter—the three judges split 2-1. Judges Lourie and Moore agreed DNA molecules could be patented, but disagreed about why and how. Judge Lourie reasoned the molecules were structurally different from those found in chromosomal DNA because covalent bonds were broken; Judge Moore found that fragments of DNA had uses that native DNA does not share, although she also noted that one reason persuading her to uphold the claims was that business decisions had rested on settled understandings that “isolated” DNA is patentable subject matter. She indicated her decision might be different had not so many claims of this type been granted over three decades.

Judge Bryson dissented. He agreed that some DNA molecules could be patented, if they were altered to a form not found in nature, such as complementary DNA (cDNA) sequences lacking introns. But he argued claims to a DNA molecule whose sequence was found in nature were not patentable. Bryson noted, “In its simplest form, the question in this case is whether an individual can obtain patent rights to a human gene. From a common-sense point of view, most observers would answer, ‘Of course not. Patents are for inventions. A human gene is not an invention’” [21] (pp. 2–3 of Bryson dissent). He went on to argue that the DNA molecules claimed were identical in sequence to those in the body, or their utility as diagnostics would be lost, and DNA molecules whose sequence was found in nature were not eligible to be patented.

### Appeal to the Supreme Court

The case was appealed to the Supreme Court, which in the meantime had ruled in *Mayo v Prometheus* (see above). The Supreme Court remanded *Myriad* to the CAFC for reconsideration in light of *Mayo.* The CAFC panel reaffirmed its previous decision, with the same 2-1 split over whether DNA molecule claims were patent-eligible. The Supreme Court agreed to hear another appeal in its 2012–2013 term, considering just one four-word question: “Are human genes patentable?” [22]. Oral arguments took place on April 15, 2013, and the Supreme Court handed down its ruling on June 13 [23••].

Judge Clarence Thomas wrote the unanimous (9-0) opinion, which ruled that “A naturally occurring DNA segment is a product of nature and not patent-eligible merely because it has been isolated, but cDNA is patent-eligible because it is not naturally occurring” [23••] (p. 2). The Supreme Court’s arguments tracked closely to CAFC Judge Bryson’s dissent, and friend-of-the-court briefs filed by the US Solicitor General on behalf of the US Government, which argued that cDNA was patent-eligible, but DNA molecules corresponding to sequences found in a genome were not [24, 25].

### Litigation Since the Supreme Court Decision

The Supreme Court decision did not end the litigation battles over *BRCA* testing. On the day of the ruling, Ambry Genetics and Gene by Gene both announced they were offering *BRCA* testing services. Other laboratories indicated they intended to do so. Many laboratories now offer BRCA testing in the United States (see Table 1).

**Table 1:** BRCA1/2 testing after the Supreme Court ruling in *Myriad*

Company	Test	Cost ($)	Test description	Turn around time (TAT)	Genes analyzed	Method & other notes
Myriad Genetics 1996	Integrated/comprehensive BRACAnalysis®	$4,040	Complete *BRCA1/2* sequence; 5 common large rearrangements	14 days	*BRCA1 & BRCA2*	Methods: Multiplexed quantitative PCR and microarray-CGH analysis.
	BRACAnalysis® (not comprehensive)	$3,340	Now recommending comprehensive BRACAnalysis® for most patients	14 days	*BRCA1 & BRCA2*	
	BRCA1 or BRCA2 single site BRACAnalysis®	$475	Known familial mutations in *BRCA1* or *BRCA2*	14 days	BRCA1 & BRCA2	
	Multisite BRACAnalysis®	$575	Three Ashkenazi Jewish founder mutations	14 days	*BRCA1 & BRCA2*: c.68_69delAG [185delAG], c.5266dupC [5382insC], and c.5946delT [6174delT]	
	BRACAnalysis large rearrangement test (BART)	$700		14 days	BRCA1 & BRCA2	
	MyRisk®	B/w $4,000 and $4,500	25 genes for 8 hereditary cancers (breast, ovarian, gastric, colorectal, pancreatic, melanoma, prostate, and endometrial). *Expected to be released in the fall of 2014.*		BRCA1/2, MLH1, MLH2, PMS2, EPCAM, APC, MUTYH, CDKN2A, PALB2, STX11, PTEN, TP53, CDH1, BMPR1A, SMAD4, ATM, BARD1, BRIP1, CDK4, CHEK2, NBN, RAD51C, RAD51D	
UCLA Diagnostic Molecular Pathology Laboratory May 2012	BRCA1 & 2 Ashkenazi Jewish Mutations	Approximately $500	Three Ashkenazi Jewish founder mutations	3–28 days	BRCA1 & BRCA2: c.68_69delAG [185delAG], c.5266dupC [5382insC], and c.5946delT [6174delT]	Methods: Sanger sequencing. Note: Send out other samples to Myriad; only offer the Ashkenazi panel.
Ambry Genetics June 13, 2014	*BRCA1 or BRCA2* site specific analysis	$400	Known familial mutations in BRCA1 or BRCA2	7–14 days	*BRCA1 or BRCA2*	Methods: NGS of coding exons. Deletion/duplication analysis using the multiplex ligation-dependent probe amplification (MLPA). BRCAplus uses a custom targeted microarray to identify gross deletions or duplications. OvaNext and CancerNext use NGS or Sanger sequencing. All mutations and VUS’ are confirmed through Sanger sequencing
	*BRCA* Ashkenazi Jewish panel	$500	Three Ashkenazi Jewish founder mutations	7–10 days	BRCA1 & BRCA2: c.68_69delAG [185delAG], c.5266dupC [5382insC], and c.5946delT [6174delT]	
	*BRCA1/2* deletion/ duplication only	$500	Rearrangement analysis	14 days	BRCA1 & BRCA2	
	*BRCA1/2* sequence + deletion/duplication	$2,200	Seq. and rearrangement analysis	14–21 days (avg: 13.9)	BRCA1 & BRCA2	
	BRCA Ashkenazi Jewish 3-site mutation panel w/ reflex to *BRCA1/2* Analysis if negative	$2,250	Three founder mutations. If test comes back negative, reflex to full sequencing of *BRCA1/2*	14–21 days	BRCA1 & BRCA2: c.68_69delAG [185delAG], c.5266dupC [5382insC], and c.5946delT [6174delT]. REFLEX to sequence of BRCA1 & BRCA2	
	*BRCA1/2* analysis w/ reflex to BRCAplus if negative	$3,350	Full seq. of *BRCA1/2*. If negative, del/dup analysis of four other high-risk genes	14–21 days	BRCA1/2 with REFLEX to BRCA1/2, CDH1, PTEN, STK11 and TP53 if negative	
	*BRCA*plus®	$3,300	6-gene high risk panel; Sequence and Del/Dup analysis	21 days	BRCA1/2, CDH1, PTEN, STK11 and TP53	
	BreastNext®	$3,900	18 gene Del/Dup analysis	6–10 weeks	ATM, BARD1, BRCA1, BRCA2, BRIP1, CDH1, CHEK2, MRE11A, MUTYH, NBN, NF1, PALB2, PTEN, RAD50, RAD51C, RAD51D, STK11 and TP53	
	OvaNext®	$3,900	Full gene seq. and del/dup analysis of *23 genes* for breast, ovarian and/or uterine cancers. Specific mutation analysis available for known familial mutations	8–12 weeks	ATM, BARD1, BRCA1, BRCA2, BRIP1, CDH1, CHEK2, EPCAM, MLH1, MRE11A, MSH2, MSH6, MUTYH, NBN, NF1, PALB2, PMS2, PTEN, RAD50, RAD51C, RAD51D, STK11, and TP53.	
	CancerNext-Expanded	$4,490	Full gene seq. of 43 and dup/del analysis of 42 genes for breast, colon, ovarian, uterine and other cancers. Specific mutation analysis available for known familial mutations	8–12 weeks	APC, ATM, BARD1, BRCA1, BRCA2, BRIP1, BMPR1A, CDH1, CDK4, CDKN2A, CHEK2, EPCAM, FH, FLCN, MAX, MET, MITF, MLH1, MRE11A, MSH2, MSH6, MUTYH, NBN, NF1, PALB2, PMS2, PTEN, RAD50, RAD51C, RAD51D, RET, SDHA, SDHAF2, SDHB, SDHC, SDHD, SMAD4, STK11, TMEM127, TP53, TSC1, TSC2, and VHL	
	CancerNext®	$4,250	Full gene seq. and dup/del analysis of *28 genes* for breast, colon, ovarian, uterine and other cancers. Specific mutation analysis available for known familial mutations	8–12 weeks	APC, ATM, BARD1, BRCA1, BRCA2, BRIP1, BMPR1A, CDH1, CDK4, CDKN2A, CHEK2, EPCAM, MLH1, MRE11A, MSH2, MSH6, MUTYH, NBN, NF1, PALB2, PMS2, PTEN, RAD50, RAD51C, RAD51D, SMAD4, STK11, and TP53	
University of Washington June 14, 2014	Single gene analysis	$1,350	Sequencing for any gene, such as *BRCA1* or *BRCA2*	12 weeks	BRCA1 or BRCA2	Methods: NGS (Illumina HiSeq2000) Note: The clinical lab and the King lab are separate entities. The King Lab offers free BROCA testing for families who meet its testing criteria (only for subjects in King’s genetic research studies). The clinical lab at UW provides commercial BROCA panel testing for patients who are referred by their providers
	Known familial mutation	$450	Point mutation analysis	12 weeks	BRCA1 or BRCA2	
	BRCA1/2 Ashkenazi Jewish 3-site	n/a	Test not performed by Washington; samples are sent off to Mayo for testing and analysis	12 weeks	BRCA1 & BRCA2: c.68_69delAG [185delAG], c.5266dupC [5382insC], and c.5946delT [6174delT]	
	BRCA1/2 Complete Analysis	$2,200	Sequencing and dup/del	12 weeks	BRCA1 & BRCA2	
	BROCA - Cancer Risk Panel	$3,350	Complete sequence of genes and detection of large deletions, duplications, and mosaicism. Genes for breast or ovarian cancer, and also colorectal, endometrial, pancreatic, endocrine, or melanoma.	12 weeks	AKT1, APC, ATM, ATR, BAP1, BARD1, BMPR1A, BRCA1, BRCA2, BRIP1, CDH1, CDK4, CDKN2A, CHEK1, CHEK2, CTNNA1, FAM175A (Abraxas), GALNT12, GEN1, GREM1, HOXB13, MEN1, MLH1, MRE11A, MSH2 (+EPCAM), MSH6, MUTYH, NBN, PALB2, PIK3CA, PPM1D, PMS2, POLD1, POLE, PRSS1, PTEN, RAD50, RAD51, RAD51C, RAD51D, RET, SDHB, SDHC, SDHD, SMAD4, STK11, TP53, TP53BP1, VHL, and XRCC2	
Fulgent Therapeutics, LLC mid-June 2013	Breast Ovarian Cancer NGS Panel	$1450 (institutional) $2,900 (third party)	39 genes. Sequencing of 39 genes involved in hereditary breast and ovarian cancer predisposition	4–6 weeks	APC, ATM, ATR, AXIN2, BAP1, BARD1, BLM, BMPR1A, BRCA1, BRCA2, BRIP1, CDH1, CDK4, CDKN2A, CHEK2, CTNNB1, EPCAM, FANCC, HOXB13, MLH1, MRE11A, MSH2, MSH6, MUTYH, NBN, PALB2, PALLD, PMS2, PTEN, RAD50, RAD51, RAD51C, RAD51D, SMAD4, STK11, TP53, VHL, XRCC2, XRCC3	Methods: NGS (Illumina MiSeq®) for larger panels and now for BRCA1/2 analysis as well. Confirm with Sanger sequencing, sometimes use Ion Proton®. MLPA for del/dup. Note: If a VUS result is given, the lab will offer free of charge sequencing for any additional family members
	BRCA1 and BRCA2 deletion/duplication analysis	$500 (institutional only)		2 weeks	BRCA1 & BRCA2	
	BRCA1 and BRCA2 full gene sequence analysis	$500 (institutional only)		2 weeks	BRCA1 & BRCA2	
	Hereditary Cancer Panel (HCP)	$1450 (institutional) $2900 (third party)	Seq. (no rearrangement analysis) of all exonic and proximal intronic sequences of 112 genes involved in hereditary cancer predisposition (breast, colorectal, and cancers of the ovary, kidney, bladder, liver, stomach, gall bladder, prostate, skin, pancreas, brain and others)	4–6 weeks	APC, AIP, ATM, ATR, AXIN2, BAP1, BARD1, BLM, BMPR1A, BRCA1, BRCA2, BRIP1, BUB1B, CDH1, CDK4, CDKN1B , CDKN2A, CHEK2, CTNNB1, CYLD, DDB2, DICER1, EGFR, EGLN1, EPCAM, ERCC2, ERCC3, ERCC4, ERCC5, EXO1, EXT1, EXT2, FANCA, FANCB, FANCC, FANCD2, FANCE, FANCF, FANCG, FANCI, FANCL, FANCM, FH, FLCN, GALNT12, GPC3, HOXB13, HRAS, KIF1B, KIT, MAX, MC1R, MEN1, MET, MITF, MLH1, MPL, MRE11A, MSH2, MSH3, MSH6, MUTYH, NBN, NF1, NF2, PALB2, PDGFRA, PICALM, PMS1, PMS2, POLD1, PRKAR1A, PRKDC, PRSS1, PTCH1, PTEN, PTPN11, RAD50, RAD51, RAD51C, RAD51D, RB1, RBBP8, RBM15, RECQL4, RET, ROBO2, SBDS, SDHA, SDHAF2, SDHB, SDHC, SDHD, SLX4, SMAD4, SMARCB1, STK11, SUFU, TERT, TMEM127, TP53, TSC1, TSC2, TSHR, TYR, VHL, WRN, WT1, XPA, XPC, XRCC2, XRCC3	
Gene DX Gaithersburg, MD (Subsidiary of Bio-Reference Laboratories, Inc) August 28, 2013	BRCA1/2 sequencing	$1,850		8–10 days	BRCA1 & BRCA2	Methods: Capillary sequencing and Exon ArrayCGH. Cancer Panels use NGS (Illumina MiSeq®). Note: GeneDx will offer to test additional family members for the VUS for free upon detailed review of the clinical and family history
	BRCA1/2 Del/Dup	$1,000		8–10 days	BRCA1 & BRCA2	
	BRCA1/2 familial variant	For 1: $350 For 2: $500	Checks for known point mutations	2–3 weeks	BRCA1 & BRCA2	
	BRCA1/2 Sequencing and Del/Dup Analysis	$2,200		8–10 days	BRCA1 & BRCA2	
	BRCA1/2 Ashkenazi Founder Mutation Panel	$450		8–10 days	BRCA1 & BRCA2: c.68_69delAG [185delAG], c.5266dupC [5382insC], and c.5946delT [6174delT]	
	OncoGeneDx Comprehensive Cancer Panel	$4,530	Seq. and Del/Dup Analysis for 29 genes for: Attenuated Familial Adenomatous Polyposis (AFAP), Breast Cancer, Colorectal Cancer, Endometrial Cancer, Familial Adenomatous Polyposis (FAP), Ovarian Cancer, Pancreatic Cancer, and Uterine Cancer	4–5 weeks	APC, ATM, AXIN2, BARD1, BMPR1A, BRCA1, BRCA2, BRIP1, CDH1, CDK4, CDKN2A, CHEK2, EPCAM, FANCC, MLH1, MSH2, MSH6, MUTYH, NBN, PALB2, PMS2, PTEN, RAD51C, RAD51D, SMAD4, STK11, TP53, VHL, XRCC2	
	High/Moderate Risk Panel for Breast Cancer	$3,850	Seq. Analysis and/or Exon‐Level del/dup analysis of 20 genes for hereditary cancer	4–5 weeks	APC, ATM, BMPR1A, BRCA1, BRCA2, CDH1, CDKN2A, CHEK2, EPCAM, MLH1, MSH2, MSH6, MUTYH, PALB2, PMS2, PTEN, SMAD4, STK11, TP53, VH	
	Breast Cancer High Risk Panel	$3,700	Six genes high risk panel	21 days	BRCA1, BRCA2, CHH1, PTEN, STK11, TP53	
	Breast/Ovarian Cancer Panel	$3,850	Seq. and Del/Dup Analysis for 21 genes for breast and ovarian cancer	4–5 weeks	ATM, BARD1, BRCA1, BRCA2, BRIP1, CDH1, CHEK2, EPCAM, FANCC, MLH1, MSH2, MSH6, NBN, PALB2, PMS2, PTEN, RAD51C, RAD51D, STK11, TP53, XRCC2	
Quest Diagnostics mid-October 2013	BRCAvantage® Comprehensive Evaluation	$2,495	Detection of point mutations, deletions, duplications, and rearrangements in *BRCA1/2*	14 days	BRCA1 & BRCA2	Methods: NGS for sequencing and multiplex ligand-dependent probe amplification (MLPA) to detect deletions, duplications, and rearrangements
	BRCAvantage® Ashkenazi Jewish Screen w/ reflex to Comprehensive Evaluation	$500; then additional TBD cost	If Ashkenazi Screen is negative, BRCAvantage, Comprehensive will be performed			
	BRCAvantage® Ashkenazi Jewish Evaluation	$500	Detection of 3 founder mutations	7 days	BRCA1 & BRCA2: c.68_69delAG [185delAG], c.5266dupC [5382insC], and c.5946delT [6174delT]	
	BRCAvantage® Single Site	$500	Detection of a known familial mutation in *BRCA1/2*	14 days	BRCA1 or BRCA2	
	BRCAvantage® Rearrangement Evaluation	$500	Detection of deletions, duplications, and rearrangements in *BRCA1/2*	14 days	BRCA1 & BRCA2	
The University of Chicago Genetic Services November 20, 2013	BRCA1 and BRCA2 familial testing	$415	Testing for known familial BRCA1/2 mutations just by sequence analysis	2–4 weeks	*BRCA1 & BRCA2*	Methods: Sanger Sequencing. PCR for dup/del
	Custom mutation sequencing	$540	Seq. of any previously identified gene/ Test costs $390 for additional family members	4–6 weeks (second family member: 3–4 weeks)		
	Custom del/dup testing	$650	Custom deletion/duplication testing by quantitative PCR. Test costs $450 for additional family members	4–6 weeks (2nd family member: 3-4 weeks)	dependent	
	BRCA1 and BRCA2 founder mutations	$475		2–4 weeks	BRCA1 & BRCA2: c.68_69delAG [185delAG], c.5266dupC [5382insC], and c.5946delT [6174delT]	
Laboratory Corporation of America Holdings (LabCorp) December 2, 2013	BRCA1 targeted analysis	$500	Just sequencing	14 days	*BRCA1*	Methods: Sanger sequencing for sequencing. Multiplex ligation-dependent probe amplification (MLPA) platform for del/dup
	BRCA2 targeted analysis	$500	Just sequencing	14 days	BRCA2	
	BRCA1/2 comprehensive analysis (BRCAssure®)	$2,895	Sequencing and del/dup analysis	21 days	*BRCA1/2*	
	BRCA1/2 deletion/duplication analysis (BRCAssure®)	$700	Just del/dup analysis	14 days	*BRCA1/2*	
	BRCA1/2 Ashkenazi Jewish Profile (BRCAssure®)	$600	Just sequencing, not del/dup or rearrangements	10–12 days	BRCA1 & BRCA2: c.68_69delAG [185delAG], c.5266dupC [5382insC], and c.5946delT [6174delT]	
InVitae December 2013	Hereditary breast and ovarian cancer syndrome	$1,500	Seq. and dup/del analysis for hereditary breast and ovarian cancer	2 weeks	BRCA1 & BRCA2	Methods: NGS. Note: Invitae offers any test and every test for one price of $1,500. In addition to choosing from among their various multi-gene panels, you can design your own test
	High-Risk Hereditary Breast Cancers	$1,500	Seq. and dup/del analysis of 7 genes for: Hereditary breast and ovarian cancer syndrome, PTEN hamartoma tumor syndrome (Cowden syndrome), Li-Fraumeni syndrome, Peutz-Jeghers syndrome, Hereditary diffuse gastric cancer	2 weeks	BRCA1, BRCA2, PALB2, PTEN, TP53, STK11, CDH1	
	Women’s Hereditary Cancers	$1,500	Seq. and dup/del analysis of 17 genes for Hereditary Breast, Ovarian, and Endometrial Cancer Syndromes	2 weeks	BRCA1, BRCA2, PTEN, TP53, MLH1, MSH2, MSH6, EPCAM, PMS2, STK11, CDH1, CHEK2, RAD51C, BRIP1, PALB2, NBN, ATM	
	Hereditary Cancer Syndromes	$1,500	Seq. and dup/del analysis for 29 genes for: Hereditary breast and ovarian cancer syndrome, PTEN hamartoma tumor syndrome, Li-Fraumeni syndrome, Lynch syndrome, Familial adenomatous polyposis, Juvenile polyposis syndrome, Peutz-Jeghers syndrome, MYH-associated polyposis syndrome, Hereditary diffuse gastric cancer, Familial cutaneous melanoma, Familial pancreatic adenocarcinoma, Hereditary papillary renal cell carcinoma, Multiple endocrine neoplasia, type 1, Multiple endocrine neoplasia, type 2, Basal cell nevus syndrome, Von Hippel-Lindau syndrome, Moderate-risk breast cancer susceptibility	2 weeks	BRCA1, BRCA2, PTEN, TP53, MLH1, MSH2, MSH6, EPCAM, PMS2, APC, BMPR1A, SMAD4, STK11, MUTYH, CDH1, CDK4, CDKN2A, PALLD, MET, MEN1, RET, PTCH1, VHL, CHEK2, BRIP1, PALB2, RAD51C, NBN, ATM	
	Family Testing Services	$200	Targeted mutation analysis offered to family members of those who have received a positive result on one of the genetic tests	2 weeks	dependent	
Center for Human Genetics, Inc January 2014	Three founder mutations	$450			BRCA1 & BRCA2: c.68_69delAG [185delAG], c.5266dupC [5382insC], and c.5946delT [6174delT]	Methods: Targeted variant analysis (PCR with RFLP). Have “plans to offer full-gene seq. and dup/del analysis in the future.”
CounsylSpring 2014	Counsyl Inherited Cancer (BRCA) Screen	$999	Sequence and dup/del analysis	2 weeks	BRCA1 & BRCA2	Method: Next-gen Illumina-based assay, but custom build their own hardware and software. Note: Anyone who takes a Counsyl screen can opt for a complimentary phone consultation
University of Michigan State Testing Lab Spring 2014	Tier 1 BRCA Mutation Panel” BRCA1 & BRCA2 sequencing	$2,220		10–28 days	BRCA1 & BRCA2	Methods: Bi-directional Sanger Sequence Analysis w/ Applied Biosystems 3730 capillary sequencing instrument. Note: Require a $85 pathology interpretation, so this cost must be added onto each of the test costs listed
	Tier 2 BRCA Mutation Panel: BRCA1 & BRCA2 dup/del	$1,222		10–28 days	BRCA1 & BRCA2	
	BRCA Ashkenazi Jewish Founder Mutations	$545		10–28 days	BRCA1 & BRCA2: c.68_69delAG [185delAG], c.5266dupC [5382insC], and c.5946delT [6174delT]	
	BRCA1/2 Targeted Sequencing, Familial	BRCA1: $401. BRCA2: $401		10–28 days	BRCA1 or BRCA2	
	BRCA1/2 Gene Sequencing	BRCA1: $1,003. BRCA2: $1,367		10–28 days	BRCA1 & BRCA2	
	BRCA1/2 del/dup analysis	BRCA1: $617 BRCA2: $617		10–28 days	BRCA1 & BRCA2	
Pathway Genomics June 3, 2014	BRCATrue®	$1,799	Sequencing and rearrangement analysis. Price includes full gene sequencing and common rearrangments, but unclear if there is an additional price for uncommon rearrangements	2 weeks	BRCA1 & BRCA2	Methods: NGS and confirms any mutaitons with Sanger. “Large gene rearrangements (large deletions or duplications) within the BRCA1 and BRCA2 genes are detected using quantitative PCR (qPCR). Positive results are confirmed by array comparative genomic hybridization (aCGH).” Note: Have a “One for One” program; for every one test ordered, one is donated to a person in need
BCM Medical Genetics Laboratories Spring 2014	BRCA1 Gene Sequencing by Massively Parallel Sequencing	Institutional: $1,500. Insurance: $2,800		49 days	BRCA1	Methods: NGS sequencing. Array CGH Analysis (aCGH), and Multiplex Ligation-dependent Probe Amplification (MLPA). Payment Notes: Baylor has 2 price points, the lower prices correspond to the *Institutional* and *Self-pay* costs, while the higher prices are billed to *Insurance* companies
	BRCA1 Sequence Analysis (Familial Mutation/Variant Analysis)	Institutional: $325. Insurance: $1,300		21 days	BRCA1	
	BRCA1 Sequence Analysis (Prenatal Sequence Analysis)	Institutional: $1,400. Insurance: $3,900		21 days	BRCA1	
	BRCA2 Gene Sequencing by Massively Parallel Sequencing	Institutional: $1,500. Insurance: $3,200		49 days	BRCA2	
	BRCA2 Sequence Analysis (Familial Mutation/Variant Analysis)	Institutional: $325. Insurance: $1,300		21 days	BRCA2	
	BRCA2 Sequence Analysis (Prenatal Sequence Analysis)	Institutional: $1,400. Insurance: $3,900		21 days	BRCA2	
	BRCA1/2 Sequence & Del/Dup Analysis	Institutional: $999. Insurance: $1,499	Test is sent to Counsyl, NOT done in Baylor’s lab	14 days	BRCA1, BRCA2	
	Comprehensive Hereditary Cancer Panel	Institutional: $4,000. Insurance: $6,749	61 genes	10 weeks	ALK, APC, ATM, BARD1, BMPR1A, BRCA1, BRCA2, BRIP1, CBL, CDC73, CDH1, CDK4, CDKN1C, CDKN2A, CEBPA, CHEK2, ENG, EPCAM, FH, FLCN, GATA2, GPC3, MAX, MEN1, MET, MLH1, MRE11A, MSH2, MSH6, MUTYH, NBN, NF2, PALB2, PAX5, PHOX2B, PMS1, PMS2, PRF1, PRKAR1A, PTCH1, PTEN, PTPN11, RAD50, RAD51C, RAD51D, RET, RUNX1, SBDS, SDHA, SDHAF2, SDHB, SDHC, SDHD, SMAD4, STK11, SUFU, TMEM127, TP53, TSHR, VHL, and WT1	
	Hereditary Breast/Ovarian/ Endometrial Cancer Panel	Institutional: $3,500. Insurance: $4,949	23 genes	8 weeks	ATM, BARD1, BRCA1, BRCA2, BRIP1, CDH1, CHEK2, EPCAM, MLH1, MRE11A, MSH2, MSH6, MUTYH, NBN, PALB2, PMS1, PMS2, PTEN, RAD50, RAD51C, RAD51D, STK11, and TP53.	
	Hereditary High Risk Breast Cancer Panel	Institutional: $3,000. Insurance: $4,299	7 genes	6 weeks	BRCA1, BRCA2, CDH1, PALB2, PTEN, STK11, and TP53.	
Emory Genetics Lab Spring 2014	BRCA1/BRCA2 Gene Seq. Panel	$2,100		3 weeks	BRCA1, BRCA2	Methods. Sequencing by NGS. Sequence analysis is required before dup/del analysis (using targetted CGH array)
	BRCA1/BRCA2 Del/Dup Panel	$750		7 days		
	BRCA1/BRCA2 Gene Seq. and Del/Dup Panel	2,300		3 weeks	BRCA1, BRCA2	
	High Risk Breast Cancer: Seq. and Del/dup Panel	$2,800		4 weeks	BRCA1, BRCA2, CDH1, PALB2, PTEN, STK11, TP53	
	Breast and Ovarian Cancer: Seq. and Del/dup Panel	$2,800		4 weeks	ATM, BRCA1, BRCA2, BRIP1, CDH1, CHEK2, EPCAM, MLH1, MSH2, MSH6, MUTYH, NBN, PALB2, PMS1, PMS2, PTEN, RAD51C, RAD51D, STK11, TP53	
	Hereditary Cancer Syndrome: Sequencing Panel	$3,200		12 weeks	ALK, APC, ATM, BMPR1A, BRCA1, BRCA2, BRIP1, CDC73, CDH1, CDKN1C, CDKN2A, CHEK2, EPCAM, FH, FLCN, GPC3, MAX, MEN1, MET, MLH1, MSH2, MSH6, MUTYH, NBN, NF2, PALB2, PHOX2B, PMS1, PMS2, PRKAR1A, PTCH1, PTEN, RAD51C, RAD51D, RET, SDHAF2, SDHB, SDHC, SDHD, SMAD4, STK11, SUFU, TMEM127, TP53, VHL, WT1	

Presented in chronological order, with the first company to offer testing listed first (Myriad Genetics)

*Source* Adapted from Annie Niehaus, Pomona College. Originally prepared for her senior thesis, “Genetic Testing for Inherited Risk of Breast and Ovarian Cancers: Payment Issues following the 2013 Myriad Supreme Court Case,” Pomona College, Claremont, California, June 2014 [31]

Even before the Supreme Court decision, Myriad Genetics issued public statements that it had over 500 claims in 23 patents, most of which were not challenged by the ACLU. Myriad began filing lawsuits against firms that offered *BRCA* testing. On July 9, 2013, Myriad sued Ambry Genetics in federal district court in Utah. The next day, Myriad filed a similar suit against Gene by Gene. Myriad has since filed suits against Quest Diagnostics, Labcorp, Invitae, GeneDx, and Pathway Genomics. Some of those firms filed requests for declaratory judgment of non-infringement in California federal district courts. The pretrial proceedings have been consolidated in the Salt Lake City, Utah, court of Judge Robert Shelby, by order of a panel of judges who decide the disposition of cases that involve multiple district courts [26]. All of the cases include *BRCA* testing; some also cover patents on *MUTYH*, a gene involved in some forms of inherited colorectal and other cancers.

In February, 2014, Gene by Gene settled with Myriad, and agreed not to offer BRCA testing in the United States. In March 2014, Judge Shelby denied Myriad’s motion for a preliminary injunction against Ambry [27•]. If Judge Shelby had granted the injunction, Ambry would have had to stop offering *BRCA* testing while the litigation was underway, and Myriad’s other competitors would likely have done likewise. By denying the injunction, Ambry can remain in the market, although it could still lose the case and could be forced to pay damages to Myriad.

Judge Shelby’s ruling directly addressed the likelihood that Myriad would prevail in its various lawsuits; his ruling is skeptical that the DNA molecule claims on primers and probes, or on amplification methods, will be upheld. Judge Shelby held a case management hearing in April 2014. He dismissed Ambry’s antitrust claims against Myriad [28]. He also set a deadline for further pleadings of July 1, 2014, set a deadline for the discovery process in which litigants gather documents and other materials in February 2015, and set limits on the number and duration of categories of witnesses [29]. That is, he set rules for any subsequent trials and the discovery process for fact-finding and documentation.

In parallel, Myriad has appealed Judge Shelby’s denial of an injunction to the CAFC. If CAFC affirms Judge Shelby, then any trials would proceed while competition continues in the *BRCA* testing marketplace; if CAFC reverses Judge Shelby, then Ambry would have to exit the market, and other companies would likely to do likewise. These trials are in a race against patent expiration, with the first of the patents-in-suit expiring in August 2014 and the ten broadest patents set to expire by the end of 2015. If they go to trial and proceed through the court system, they may help to clarify the boundaries of what is patentable and what is not, and may also make public some of the history of scientific discovery of the relevant genes because of the rigorous legal discovery process and court proceedings. Finally, in August 2014, GeneDx, one of the defendants sued by Myriad for patent infringement, filed eleven petitions at the US Patent and Trademark Office, seeking reexamination of claims in 11 patents [30]. This is an administrative procedure that cannot challenge patentable subject matter, but raises questions of novelty, nonobviousness, enablement and written description. It will proceed in parallel to the litigation already underway.

## Summary and Conclusion

A series of recent Supreme Court decisions have challenged the conventional wisdom and legal expectations about what can be patented. Ongoing litigation and future cases will further refine patent law, not only what can be patented but perhaps also how criteria will be applied to determine when an invention is new, useful, nonobvious, fully enabled, and adequately described.

The technologies for measuring DNA and proteins have moved rapidly in recent decades, as have methods for accumulating, digitizing, storing, and analyzing data. The analysis of the information that results of testing is at least as complex and difficult as generating the data, and it is essential to clinical interpretation. Indeed, the profusion of data is bringing the importance of algorithms and computational methods to the fore. Such methods can also be patented, regulated, or paid for as services. The focus of this chapter, however, is on genetic tests to determine the sequence taken from a body sample.

Four main conclusions can be drawn from this history. First, the Supreme Court has made clear that patent practice for three decades has entailed granting exclusive rights that are too broad and cover products of nature and laws of nature. Two unanimous decisions about diagnostic technologies have narrowed the range of patentable subject matter of both methods and DNA molecules. Second, the boundaries of patentable subject matter are still fuzzy. The Supreme Court has unanimously ruled that lower courts were upholding patents claims they should not have upheld, but it has not indicated how to draw the line between methods and molecules that are patent-eligible and those that are not. Third, the Court’s decisions reduce the risk of infringement liability for multi-gene and whole-genome analysis. It is not clear where the line is or how many genes one needs to test to avoid infringement liability, but it is highly likely that those holding patent claims on individual genes will not be able to enforce them against whole-genome analysis. Finally, and most directly relevant to the future of genetic testing, the Supreme Court has clearly noted that it will permit patents on some DNA inventions. Such patents will not likely be on individual genes, however, but on technologies and methods that more clearly reflect the art of invention rather than the labor of discovery.

## Footnotes

1 Number of tests taken from Myriad public statement to the US Patent and Trademark Office in January 2013; revenues from 10 K and annual reports compiled by the author.

2 Interviews with Chris Hansen (ACLU), Lori Andrews (Kent School of Law), Tania Simoncelli (ACLU), and Sandra Park (ACLU) and summarized in Alexandra Young 18.Young, A., *Prelude to ‘Pigs Fly:’ The Early History of the Myriad Case,undergraduate thesis outside the disciplines*, in *Thesis Outside the Disciplines*. 2014, Duke University: Durham, NC. See also 19.Jasanoff, S., *Dis*-*Owning Nature: The BRCA Gene Patents and the Supreme Court*, in *Program on Science, Technology and Society, Harvard Kennedy School*, S. Jasanoff, Editor. 2013: Cambridge, MA.
